# Supplementary material for: Comparing rates of introgression in parasitic feather lice with differing dispersal capabilities
Source: Commun Biol. 2020 Oct 23;3:610. doi: 10.1038/s42003-020-01345-x (PMC7584577; doi:10.1038/s42003-020-01345-x)
Supplement: Supplementary file 1 — Reporting Summary [file 42003_2020_1345_MOESM1_ESM.pdf]

nature research

Corresponding author(s): Jorge Doña

Last updated by author(s): Sep 11, 2020

Reporting Summary

Nature Research wishes to improve the reproducibility of the work that we publish. This form provides structure for consistency and transparency in reporting. For further information on Nature Research policies, see Authors & Referees and the Editorial Policy Checklist .

Statistics

For all statistical analyses, confirm that the following items are present in the figure legend, table legend, main text, or Methods section.

n/a	Confirmed	
☐	☒	The exact sample size (*n*) for each experimental group/condition, given as a discrete number and unit of measurement
☒	☐	A statement on whether measurements were taken from distinct samples or whether the same sample was measured repeatedly
☐	☒	The statistical test(s) used AND whether they are one- or two-sided<br>*Only common tests should be described solely by name; describe more complex techniques in the Methods section.*
☐	☒	A description of all covariates tested
☒	☐	A description of any assumptions or corrections, such as tests of normality and adjustment for multiple comparisons
☐	☒	A full description of the statistical parameters including central tendency (e.g. means) or other basic estimates (e.g. regression coefficient) AND variation (e.g. standard deviation) or associated estimates of uncertainty (e.g. confidence intervals)
☐	☒	For null hypothesis testing, the test statistic (e.g. *F*, *t*, *r*) with confidence intervals, effect sizes, degrees of freedom and *P* value noted<br>*Give P values as exact values whenever suitable.*
☒	☐	For Bayesian analysis, information on the choice of priors and Markov chain Monte Carlo settings
☒	☐	For hierarchical and complex designs, identification of the appropriate level for tests and full reporting of outcomes
☒	☐	Estimates of effect sizes (e.g. Cohen's *d*, Pearson's *r*), indicating how they were calculated

Our web collection on statistics for biologists contains articles on many of the points above.

Software and code

Policy information about availability of computer code

Data collection	N/A
Data analysis	Atram 1.0, fastqSplitDups, Fastx_clipper v0.014, Fastx_trimmer v0.014, Fastq_quality_trimmer v0.014, Fastqc v0.11.5, Phylonet v3.6.8, MAFFT v7.310, R v3.5.1, Phytools v0.6, RAxML v8.1.3, SPAdes v3.12.0, spplDer.

For manuscripts utilizing custom algorithms or software that are central to the research but not yet described in published literature, software must be made available to editors/reviewers. We strongly encourage code deposition in a community repository (e.g. GitHub). See the Nature Research guidelines for submitting code & software for further information.

Data

Policy information about availability of data

All manuscripts must include a data availability statement. This statement should provide the following information, where applicable:

- Accession codes, unique identifiers, or web links for publicly available datasets
- A list of figures that have associated raw data
- A description of any restrictions on data availability

All data needed to evaluate the conclusions in the paper are present in the paper and/or the Supplementary Materials. Raw sequence data, along with sequencing statistics, are available through the NCBI. Specifically, the SRA, Biosample, and BioProject accession codes (along with sequencing statistics) can be found in the supplemental table S2. Supplementary data are also available at Figshare (DOI: 10.6084/m9.figshare.9176204).

Field-specific reporting

Please select the one below that is the best fit for your research. If you are not sure, read the appropriate sections before making your selection.

Ecological, evolutionary & environmental sciences study design

All studies must disclose on these points even when the disclosure is negative.

Study description	We quantified the amount of introgression in feather lice
Research sample	71 louse individuals belonging to five taxa of wing lice (Columbicola) and seven taxa of body lice (Physconelloides)
Sampling strategy	Data represent all described New World ground-dove wing and body louse species, most host species in this group, including samples across multiple biogeographic areas within species.
Data collection	Illumina whole-genome sequence data produced at W.M. Keck Center at the UIUC. Data were available from previous studies (see Materials and Methods: Data section)
Timing and spatial scale	N/A
Data exclusions	N/A
Reproducibility	N/A
Randomization	N/A
Blinding	N/A

Did the study involve field work? ☐ Yes ☒ No

Reporting for specific materials, systems and methods

We require information from authors about some types of materials, experimental systems and methods used in many studies. Here, indicate whether each material, system or method listed is relevant to your study. If you are not sure if a list item applies to your research, read the appropriate section before selecting a response.

Materials & experimental systems

n/a	Involved in the study
☒	☐ Antibodies
☒	☐ Eukaryotic cell lines
☒	☐ Palaeontology
☒	☐ Animals and other organisms
☒	☐ Human research participants
☒	☐ Clinical data

Methods

n/a	Involved in the study
☒	☐ ChIP-seq
☒	☐ Flow cytometry
☒	☐ MRI-based neuroimaging
